# OBJECTIVE SLEEP QUALITY ASSOCIATES WITH PLASMA BIOMARKER FOR COGNITIVE IMPAIRMENT

**DOI:** 10.1093/geroni/igae098.2234

**Published:** 2024-12-31

**Authors:** Jinyoung Kim, Kevin Dangler, Stephanie Nam, Miguel Fudolig, Jefferson Kinney

**Affiliations:** University of Nevada Las Vegas, Las Vegas, Nevada, United States; University of Nevada Las Vegas, Las Vegas, Nevada, United States; University of Nevada Las Vegas, Las Vegas, Nevada, United States; University of Nevada Las Vegas, Las Vegas, Nevada, United States; University of Nevada Las Vegas, Las Vegas, Nevada, United States

## Abstract

**Aim:**

Emerging evidence suggests that poor sleep (i.e., lack of sleep and low sleep quality) significantly impacts the risk of severe cognitive impairment. However, research on the use of sleep quality metrics as early predictors of cognitive impairment risk remains scarce. In this study, we investigated the association between objective sleep quality measures and plasma levels of Eotaxin-1, an early biomarker for cognitive impairment, among nurses.

**Methods:**

We used data from 35 nurses who underwent multiple home sleep studies with electroencephalograms on workdays and days off were included. Objective sleep quality measurements included percentage of stage-3 non-rapid eye movement sleep in total sleep time (%N3) and wake time after sleep onset (WASO). Blood samples were collected in all participants and plasma levels of multiple cytokines (27Plex Cytokine array), including Eotaxin-1 were measured by Luminex multiplexing.

**Results:**

Sleep quality indicators showed that WASO increased by 8% on days off compared to workdays, while %N3 decreased by 1% (p<.001). After adjusting for age, sex, shift, and BMI, higher WASO (β=1.22, p<.05) and lower %N3 (β=-5.09, p<.05) on workdays were significantly associated with increasing levels of Eotaxin-1. Sleep parameters on days off did not significantly correlate with Eotaxin-1 levels.

**Conclusion:**

Our findings indicate a correlation between objective sleep quality measurements on workdays and an early biomarker of cognitive impairment. Further research is necessary to elucidate the underlying mechanisms linking sleep quality to future cognitive impairment, as well as to explore the potential differential associations of these parameters between workdays and days off.

